# Native human autoantibodies targeting GIPC1 identify differential expression in malignant tumors of the breast and ovary

**DOI:** 10.1186/1471-2407-8-247

**Published:** 2008-08-24

**Authors:** Victoria Yavelsky, Sarit Rohkin, Ruthy Shaco-Levy, Alina Tzikinovsky, Tamar Amir, Hila Kohn, Berta Delgado, Alex Rabinovich, Benjamin Piura, Gerald Chan, Gavreel Kalantarov, Ilya Trakht, Leslie Lobel

**Affiliations:** 1 Department of Virology, Faculty of Health Sciences, Ben Gurion University of the Negev, Beer Sheva 84105, Israel; 2 Department of Pathology and Soroka University Medical Center and Faculty of Health Sciences, Ben Gurion University of the Negev, Beer-Sheva, Israel; 3 Unit of Gynecologic Oncology, Soroka University Medical Center and Faculty of Health Sciences, Ben Gurion University of the Negev, Beer-Sheva, Israel; 4 The Morningside Foundation, 1188 Centre Street, Newton Centre, MA 02459, USA; 5 Department of Medicine, College of Physicians and Surgeons, 630 West 168th St. Columbia University, New York, N.Y. 10032, USA

## Abstract

**Background:**

We have been studying the native humoral immune response to cancer and have isolated a library of fully human autoantibodies to a variety of malignancies. We previously described the isolation and characterization of two fully human monoclonal antibodies, 27.F7 and 27.B1, from breast cancer patients that target the protein known as GIPC1, an accessory PDZ-domain binding protein involved in regulation of G-protein signaling. Human monoclonal antibodies, 27.F7 and 27.B1, to GIPC1 demonstrate specific binding to malignant breast cancer tissue with no reactivity with normal breast tissue.

**Methods:**

The current study employs cELISA, flow cytometry, Western blot analysis as well as immunocytochemistry, and immunohistochemistry. Data is analyzed statistically with the Fisher one-tail and two-tail tests for two independent samples.

**Results:**

By screening several other cancer cell lines with 27.F7 and 27.B1 we found consistently strong staining of other human cancer cell lines including SKOV-3 (an ovarian cancer cell line). To further clarify the association of GIPC1 with breast and ovarian cancer we carefully studied 27.F7 and 27.B1 using immunocytochemical and immunohistochemical techniques. An immunohistochemical study of normal ovarian tissue, benign, borderline and malignant ovarian serous tumors, and different types of breast cancer revealed high expression of GIPC1 protein in neoplastic cells. Interestingly, antibodies 27.F7 and 27.B1 demonstrate differential staining of borderline ovarian tumors. Examination of different types of breast cancer demonstrates that the level of GIPC1 expression depends on tumor invasiveness and displays a higher expression than in benign tumors.

**Conclusion:**

The present pilot study demonstrates that the GIPC1 protein is overexpressed in ovarian and breast cancer, which may provide an important diagnostic and prognostic marker and will constitute the basis for further study of the role that this protein plays in malignant diseases. In addition, this study suggests that human monoclonal antibodies 27.F7 and 27.B1 should be further evaluated as potential diagnostic tools.

## Background

We previously described the isolation and characterization of a large panel of fully human monoclonal antibodies from patients with breast cancer [1]. Many of these antibodies are highly sensitive and specific for breast cancer and some also demonstrate high sensitivity and specificity for non-autologous malignancies of different types. The antigen target of two of these antibodies, 27.F7 and 27.B1 is the protein GIPC1, which is a member of a family of PDZ-domain conserved proteins.

GIPC1 is a carboxy-terminal GAIP interacting protein and together they are components of a G-protein-coupled signaling complex thought to be involved in vesicular trafficking. The PDZ domain of the GIPC family proteins interacts with C terminal regions of FZD3, IGF1 receptor, TrkA, TGF-β RIII, integrin α6A, 5T4 and RGS19 [2]. Thus GIPC1, like other PDZ domain-containing proteins, may function to cluster signaling molecules and membrane receptors in specific membrane microdomains [3]. Because RGS19 is a member of the RGS family that regulates heterotrimeric G-protein signaling, the GIPC1 family of proteins might function as scaffolds linking heterotrimeric G-proteins to receptor tyrosine kinases.

It is also known that GIPC1 not only interacts with TGF-β type III receptor (TGF-β RIII) [4], but also induces its increased expression on the cell surface, leading to an enhanced responsiveness to TGFβ. Down-regulation of GIPC1 mRNA in tumors might promote cellular proliferation through interference of TGFβ signaling [5]. On the other hand, Awan et.al. suggested a metastatic role for GIPC1 protein demonstrating its close interaction with 5T4 protein, which has a great impact on the actin cytoskeleton and cell migration [6]. In addition, GIPC1 was shown to interact with alpha-actinin-1 [7], which is important for stabilizing actin bundles. It was also shown to be involved with cell adhesion through its close link with E-cadherin in epithelial cells [8]. Therefore, GIPC1 might play key roles in carcinogenesis and embryogenesis through modulation of growth factor signaling

In our previous study antibodies 27.F7 and 27.B1 were studied using immunofluorescence on breast cancer specimens. They were highly specific for breast cancer and did not stain normal breast tissue. To provide a more in depth analysis of these antibodies and to further clarify the association of GIPC1 with different types of breast cancer, we carefully studied 27.F7 and 27.B1 antibodies via immunohistochemical analysis of breast cancer tissue. In addition, we determined that these antibodies stained the ovarian cancer cell line SKOV-3 quite strongly and as a result we also performed a similar analysis on serous carcinoma of the ovary, the most common and aggressive type of ovarian malignancy.

Breast cancer claims the lives of many women yearly. Recently, there have been improvements in breast cancer treatment and survival, which has rested to a large extent on detection and treatment of early stage disease [9-11]. Although mammography has been quite successful in detection of breast cancer, there are still many women that die as a result of identification of the malignancy at a late stage [10-13]. Ovarian cancer, on the other hand, in particular epithelial carcinoma of the ovary is the leading cause of death from gynecologic cancers in the United States, and is the fifth leading cause of cancer death among U.S. women. It usually occurs in women over the age of 35, with most affected women being above the age of 50 [14].

Approximately 5% to 10% of ovarian cancers are familial and in most families affected with breast and ovarian cancer a genetic linkage has been established with the *BRCA1 *locus. *BRCA2*, is also responsible for some instances of inherited ovarian and breast cancer [9]. Although treatment modalities for ovarian cancer are lacking, survival in most cases seems to depend on early detection and treatment [14]. One of the principal reasons for such a high mortality is the lack of effective and reliable methods for early diagnosis of the disease [15]. Because ovarian cancer is often asymptomatic in its early stages, most patients have widespread disease at the time of diagnosis. Partly as a result of this, yearly mortality in ovarian cancer is approximately 65% of the incidence rate [16]. In addition, most diagnostic tests including manual examination and transvaginal ultrasound cannot reliably predict early onset of malignancy [17]. For early cancer screening physicians have relied, for the most part, on a variety of cancer markers such as CA-125. Many of these, however, are not reliable, as evidenced by the fallibility of CA-125 in predicting ovarian cancer, since many women with low values have malignancy [12,18].

As early detection of ovarian cancer is a key factor for long-term survival, it is an imperative that new markers for early onset ovarian cancer be developed. Since we identified GIPC1 as a new tumor-associated antigen that is linked to breast cancer and that stains ovarian cancer cells *in vitro*, we reasoned that it might be useful as a new marker for these malignancies. Therefore, we extended our studies of GIPC1 in breast and ovarian cancer to determine the utility of this cancer-associated antigen as a marker for malignancy. In particular, a study of GIPC1 protein expression in malignant cells of ovarian tumors might provide new avenues for the diagnosis, prognosis and treatment of ovarian cancer, and might constitute the basis for further study of the role that this protein might play in malignant disease.

## Methods

### Cell culture and antibodies

Human breast cancer cells MCF-7, ovarian cancer cells SKOV3 and human primary fibroblasts were obtained from the ATCC and maintained according to the supplier's instructions. Hybridoma cell line, secreting 27.F7 and 27.B1 antibodies, were produced as a result of fusion between the MFP-2 fusion partner cell line and human lymphocytes derived from lymph node lymphocytes of breast cancer patients [1]. They were maintained in 24-well plates in RPMI 1640 media (Gibco), supplemented with 10% Fetal Calf Serum, L-glutamine, non-essential amino acids, pyruvate, vitamins and hypoxanthine and thymidine (HT).

Human anti-GIPC1 monoclonal antibodies 27.B1 and 27.F7 are both IgM,κ, (Rudchenko et al., manuscript in press BMC cancer), and were produced by growing hybridoma cells and used as culture media supernatant with defined antibody concentration. For some experiments antibodies were further purifed by gel filtration chromatography on a 1 meter × 2.5 cm column packed with Sephacryl S300 in sodium phosphate buffer (pH 7.8), 300 mM NaCl at a flow rate of 6 mL/minute. Normal goat serum used for blocking was purchased from Sigma (G9023). Monovalent Fab Fragment Goat Anti-Human IgM+IgG, used for secondary blocking of light chains, was purchased from Jackson Immunoresearch Laboratories, Inc. (109-006044). ELISA capturing antibody, for determination of 27.B1 and 27.F7 antibody concentrations, goat anti-human IgM, Fc-specific, was purchased from Jackson Immunoresearch Laboratories (109007043). Peroxidase labeled goat anti-human IgM for ELISA was purchased from Sigma (A-8400). Secondary goat anti-Human kappa light chain FITC conjugated was purchased from Enco Scientific Services, (2060-02), and biotinylated goat anti-human kappa light chain, affinity purified, was purchased from Vector Laboratories, Inc. (BA-3060, P1216).

### Human tissues

The paraffin-embedded samples of biopsies of normal ovaries, ovarian and breast tumor tissues were obtained from the archives of the Institute of Pathology of Soroka Medical Center, Israel. The research was approved by the Institutional Ethics Committee. Classification of tumor samples according to clinical staging, differentiation state and tumor type was performed by the experienced pathologists from The Institute of Pathology, Soroka Medical Center.

### Cellular ELISA (cELISA)

#### Preparation of fixed target cells for cELISA antibody screening

Upon reaching 80–90% confluency, the target cells were detached from the plastic dishes by incubation with 0.02% ethylenediaminetetraacetic acid (EDTA) and 0.25% trypsin for 5 min at 37°C. After a few washes with phosphate-buffered saline (PBS) the cells were exposed to 4% formaldehyde for 15 min at room temperature and then stored at 4°C for up to 4 weeks.

#### cELISA on fixed cells

Millipore suction plates were blocked with 0.3% dry milk in PBS for 1 h at room temperature. Fixed cells were applied to each well at 5 × 10^3^–10^4 ^cells per well. Before application the fixed cells were washed and resuspended in 0.1% Tween-20/PBS (to perforate cell membranes) and incubated for 30 min at 37°C. All the following steps were performed in the presence of 0.3% milk in PBS. After applying cells to the wells and washing them at least twice by applying vacuum to the plate, antibodies were applied to the wells and incubated with the cells for 2 h at room temperature. Following removal of the antibodies and subsequent washes, secondary antibodies conjugated with HRP were applied. Conjugates used were Goat anti-Human Ig Kappa (27.F7 and 27.B1 are IgM κ). After 30 min of incubation followed by several washings, the orthophenylendiamine (OPD) substrate was added and the color intensity at 492 nm was recorded.

### Western blotting

Cells were lysed with freshly prepared ice cold lysis buffer [20 mM Tris-HCl, pH 7.6, 420 mM NaCl, 0.25% NP40, 2 mM phenylmethylsulfonyl fluoride, 1 ug/ml leupeptin, 250 U/ml Trasylol (aprotinin)] and stored at -80°C or used immediately. Protein concentration was determined with the BioRad Protein Detection Reagent (BioRad). Equal amounts of protein were separated on 10% SDS polyacrylamide gels, transferred to a nitrocellulose membrane and probed with relevant primary and HRP-conjugated secondary antibody. Membranes were processed using an enhanced chemiluminescence kit (ECL, Amersham), and visualized on Kodak BioMax MR-1 film.

### Reactivity of 27.F7 and 27.B1 monoclonal antibodies against SK-BR-3 cell line by flow cytometry

Purified 27.B1 and 27.F7 antibodies were tested against the representative tumor cell line to determine the reactivity by flow cytometry. Briefly, SK-BR-3 cells (0.9 × 10^6^/300 μL) were incubated with purified antibodies (27.B1 and 27.F7) or control human myeloma IgM at 50 μg/mL for 2 hours on ice. After incubation, the cells were washed with PBS-5% FBS and incubated with biotin-conjugated anti-human-IgM (Pierce cat #31778, diluted 1:100) for 1 hour on ice. The cells were washed with PBS-5% FBS, followed by incubation with Streptavidin-Cy-Chrome (Pharmingen cat# 13038A, diluted 1:120) for 30 minutes on ice. Finally, the cells were washed and resuspended in 0.5 mL of buffer containing propidium iodide (Molecular Probes cat# P-1304) at 0.6 μg/mL. Tumor cell binding was determined using a FACSCalibur. Antibodies were considered positive if antibody-treated tumor cells exhibited a positive shift in fluorescence of 30% or more of the cell population as compared to the negative control.

### Immunocytofluorescence

Microscope slides were treated with 1 M hydrochloric acid (HCl) to obtain proper cell adhesion, rinsed thoroughly with DI water and autoclaved. The SKOV-3, MCF-7 and SKBR-3 cell lines were trypsinized using a 0.25% trypsin solution, transferred to the slides and incubated overnight in growth media. After the incubation, immunofluorescent staining of the cells was performed, using primary 27.F7 and 27.B1 human antibodies for GIPC1 detection. SKOV-3, SKBR-3, MCF-7 cells were stained according to the following immunofluorescence staining protocol. Briefly, slides were washed with PBS and blocked using blocking solution (5% normal goat serum) (Sigma, G9023) in PBS. After a series of washings (3 times in PBS, 5 minutes each), the slides were covered with 2 micrograms/ml of primary human monoclonal antibody 27.F7 or 27.B1 (IgM, k), for 1 hour at room temperature in a humid chamber. After the incubation, the slides were washed in PBS, blocked as above, and incubated with a secondary anti-human kappa light chain FITC conjugated antibodies (Enco Scientific Services, 2060-02) for 30 minutes. The slides were washed several times, drained, mounted with mounting medium (Biomedia, M01) and coverslips were applied. Cell staining was then examined by fluorescence microscopy.

### Antigen blocking

GIPC1 protein was expressed in bacteria with a 6× histidine tag, purified in denatured form by NTA resin chromatography (Qiagen), and refolded as previously described [19]. GIPC1 protein (15 micrograms/mL final concentration in blocking solution) was preincubated with 27.F7/27.B1 antibody (1.5 micrograms/mL final concentration in blocking solution) for 1 hr at RT. Samples of breast malignant tissue and an ovarian malignant tumor, which were previously found positive for GIPC1 staining, were utilized for this analysis. Normal ovary and normal breast tissue served as negative controls. The staining procedure was performed according to the immunohistochemistry protocol outlined below, except that instead of applying primary antibody alone a mixture of antibody preincubated with GIPC1 protein was applied to the slides during staining. The results were examined by light microscopy.

### Immunohistochemistry

Five μm sections were obtained from paraffin blocks. Endogenous peroxidase activity was blocked by incubation of slides in 3% H_2_O_2 _in methanol. Following washing, tissue slides were blocked with 5% normal goat serum in PBS. Monovalent Fab fragments of goat anti-human IgM+IgG (Jackson Immunoresearch Laboratories, Inc.), in blocking solution, was then applied for secondary blocking. The slides were washed 3 times in PBS and incubated with primary human monoclonal antibody 27.F7 or 27.B1 in blocking solution. The slides were then washed and incubated with a secondary antibody. The next steps in the staining procedure were performed using VECTASTAIN^® ^ABC KIT (Standard) (Vector Laboratories Inc.) according to the recommended protocol. The slides were then incubated with the Diaminobenzidine peroxidase substrate (DAB) (Sigma FAST™ 3,3 Diaminobenzidine tablet sets). Mayer's Hematoxylin was applied for nuclear counterstaining. The slides were subsequently washed several times, drained, dehydrated, and mounted with Eukitt^® ^quick-hardening mounting medium and covered with coverslips. For negative controls, the primary antibody was replaced by phosphate-buffered saline in each set of staining.

### Immunohistochemical analysis

The sections were reviewed by two pathologists (R.S.L and B.D). Extent and intensity of staining were evaluated. Extent assessed roughly how much of the pertinent area in the tissue was stained and was scored as percentages: 0%, 10%, 20%, 30%, etc. up to 100% immunoreactive epithelial cells. Intensity assessed the strength of staining and was scored as 1+, weak; 2+, moderate; and 3+, strong staining. Only moderate and strong staining, exceeding the background staining, observed in ≥ 10% of the section was considered positive. Such staining was not observed in the non-neoplastic tissue in tumor sections.

### Statistical analysis

Statistical analysis using Fisher one-tail and two-tail tests for two independent samples was performed, testing the significance of the difference between 27.B1 and 27.F7 antibodies in their ability to detect positive GIPC1 cases in different malignant and benign tumors of the ovary and breast. This test was also used to make comparisons between different tumors with respect to the frequency of positive cases detected by these antibodies. The P value indicates significance at a value of < 0.05.

## Results

### Fully human monoclonal antibodies 27.B1 and 27.F7 detect GIPC1 in different cancer cell lines in a highly specific manner

We previously described the construction of a unique fusion partner cell line, MFP-2, and its use for the immortalization of both human peripheral blood and lymph node B-lymphocytes [1]. We have also demonstrated that MFP-2 was employed for the isolation of a panel of autologous breast cancer specific antibodies from breast cancer patients [20]. Two of these native fully human monoclonal antibodies (fhMAbs), designated 27.B1 and 27.F7, derived from lymph node B-cells of a breast cancer patient, whose target is the PDZ domain-containing protein known as GIPC1, were chosen for further study. We previously determined that this protein is specifically up-regulated in malignant breast epithelial tissue/cells and in breast cancer cell lines, is not detected in normal breast epithelia, and the cytosol/membrane localization of the target antigen is especially strong (Rudchenko et al., manuscript in press BMC cancer).

**Figure 1:**
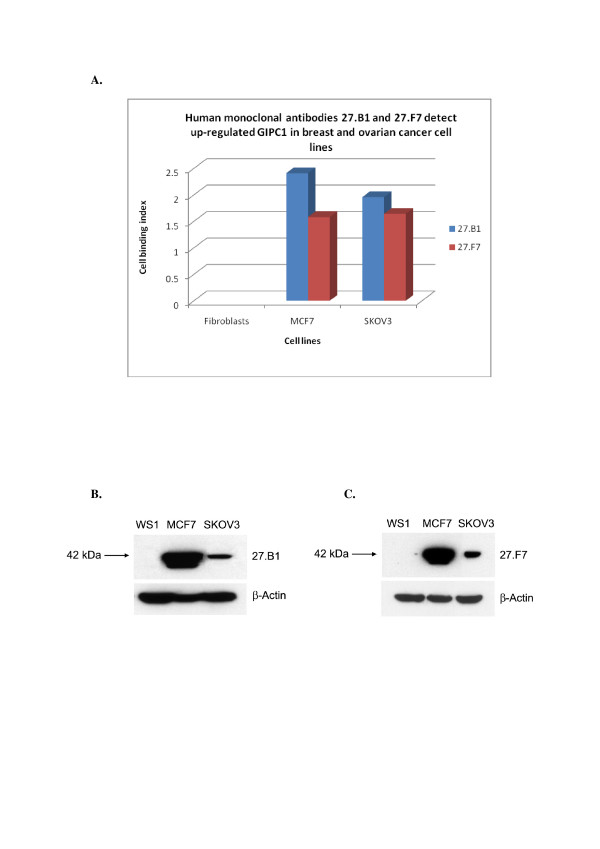
**Immunoreactivity of human monoclonal antibodies (hMAbs) 27.B1 and 27.F7 against different cancer cell lines**. A, cELISA: The binding index is the normalized antibody binding relative to the background antibody reactivity with primary human fibroblasts. Both hMAbs 27.B1 and 27.F7 display an increased binding to formalin-fixed breast (MCF7) and ovarian (SKOV3) cancer cell lines in comparison to normal fibroblasts (WS1). B, and C, Western blot analysis: The target antigen, GIPC1, for monoclonal 27.B1 (B) and 27.F7 (C) in MCF7 and SKOV3 cancer cell lines was identified by Western blot. The target antigen is present in MCF7 (breast) and SKOV3 (ovarian) cell lines but is not detected in WS1 (normal fibroblasts). β-Actin served as the loading control.

The question that arises based on our previous results described above is whether fhMAbs 27.B1 and 27.F7 react specifically with breast cancer only, or this reaction is characteristic of other neoplasias. To investigate this, we tested the 27.F7 and 27.B1 antibodies using cELISA, Western blot analysis and immunocytofluorescent staining on several other cell lines, including the already examined MCF-7 breast cancer cell line and the additional cell line SKOV-3 (ovarian carcinoma), thus expanding the analysis to other cell types, using WS1 (human primary fibroblasts) as a negative control. The results have revealed that both 27.B1 and 27.F7 antibodies demonstrate positive immunoreactivity against breast (MCF7) and ovarian (SKOV3) cancer cell lines (Figure 1). Immunofluorescent analysis demonstrated an apparent diffuse cytoplasmic staining of MCF-7 and SKOV-3 cancer cells, whereas human fibroblasts had no detectable staining (Data not shown).

### Fully human monoclonal antibodies 27.F7, 27.B1 react with the cell surface of SKBR-3

**Figure 2:**
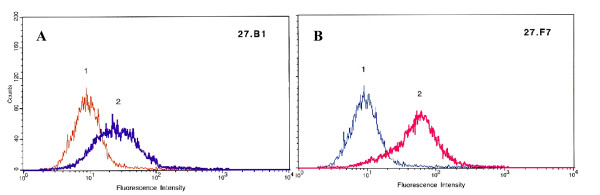
**Reactivity of 27.B1 (A) and 27.F7 (B) against SK-BR-3**. Fluorescence intensity of tumor cells incubated with myeloma IgM (1), 27.B1 (A-2), and 27.F7 (B-2) demonstrate positive cell-surface reactivity against the breast carcinoma cell line, SK-BR-3.

To evaluate the relative affinity of the 27.F7 and 27.B1 human monoclonal antibodies to breast cancer cells we employed flow cytometry with live SKBR-3 cells. The results showed that both 27.B1 and 27.F7 antibodies demonstrate positive cell-surface reactivity against the breast cancer cell line, SKBR-3. The antibody 27.F7 demonstrated strong reactivity with an 8.5-fold increase in median fluorescence (MF) above the negative control (myeloma IgM), whereas antibody 27.B1 displayed a lower median fluorescence with a 3.7-fold increase in MF over the negative control. Representative flow histograms are shown in Figure 2. These results demonstrate that these two antibodies display a differential binding pattern to GIPC1 antigen in live cancer cells.

### Internalization of 27.B1 and 27.F7 monoclonal antibodies into breast cancer cells

**Figure 3:**
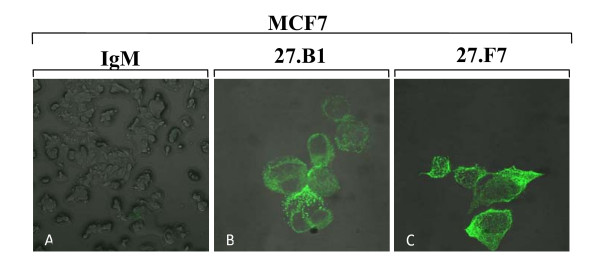
**Internalization of 27.B1 and 27.F7 antibodies into breast cancer cells**. 27.B1(B) and 27.F7 (C) antibodies were internalized into MCF7 cells. Antibody-treated cells were evaluated for intracellular staining with the aid of laser scanning confocal microscopy. Warming of the antibody-bound cells to 37°C for 30 minutes revealed intracellular staining of the MCF7 breast cancer cell line. In contrast, no intracellular staining was visualized with the non-internalizing control antibody (human myeloma IgM).

Following the results demonstrated positive cell-surface reactivity of both 27.B1 and 27.F7 antibodies against the breast cancer cell line, SKBR-3, we tried to determine if the cell-surface bound antibodies were internalized into MCF7 cells rather than shed from the plasma membrane. To this end, antibody-treated live cells were further evaluated for intracellular staining by confocal microscopy. The results demonstrated that both 27.B1 (Figure 3B) and 27.F7 (Figure 3C) antibodies demonstrated positive intracellular staining of MCF7 breast cancer cell line. In contrast, clear intracellular staining was not visualized with the non-internalizing control antibody (human myeloma IgM) (Figure 3A).

### 27.B1 and 27.F7 monoclonal antibodies specifically bind GIPC1 antigen in paraffin-embedded tissue samples

To confirm the specificity of 27.F7 and 27.B1 antibodies to the native GIPC1 protein, we performed an antigen blocking experiment. For this purpose, 27.F7 and 27.B1 antibodies were incubated with a high concentration of bacterial expressed and refolded GIPC1 antigen [19] prior to application on tissues, and then the standard protocol (see Materials and Methods) for immunohistochemical staining of human tissues was employed. We used specimens from a paraffin-embedded tissue block that were previously stained with these antibodies and identified as positive. Following an antibody-blocking procedure for endogenous immunoglobulins in the tissue, the specimens were examined by light microscopy, and no staining was detected: 27.B1 and 27.F7 antibodies were both blocked completely following the addition of exogenous GIPC1 antigen whereas addition of extraneous protein, such as lysozyme, did not block antibody staining (data not shown). This confirmed the specificity of these antibodies to the GIPC1 antigen in the tissue sections. These results demonstrate that monoclonal antibodies 27.B1 and 27.F7 target endogenous GIPC1 protein specifically, and therefore relative tissue staining likely reflects the cellular expression level of the GIPC1 protein.

### Human monoclonal antibodies 27.B1 and 27.F7 identify elevated GIPC1 protein levels in breast cancer with differential staining patterns that correlate with tumor invasiveness

**Figure 4:**
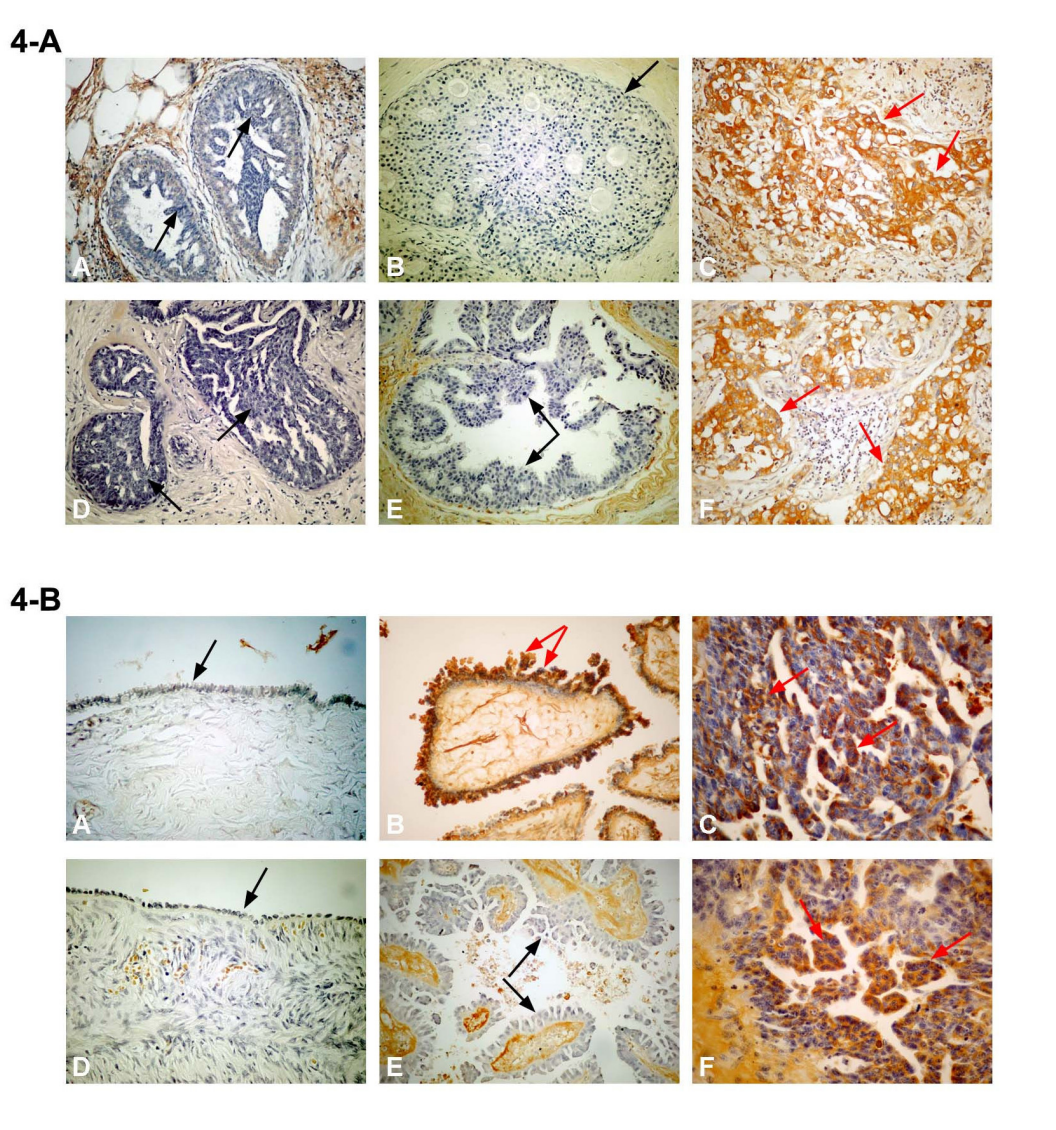
**Immunohistochemistry of ovarian and breast tissues**. **4-A **Immunohistochemistry of breast tumor tissue using 27.B1 (A, B, C) and 27.F7 (D, E, F) antibodies. Hyperplasia (A, D) and ductal carcinoma in situ (B, E) are negative, and invasive ductal carcinoma (C, F) is positive (Original magnification ×200). Black arrows indicate negative stained regions and red arrows indicate positive stained regions. **4-B **Immunohistochemistry of ovarian tissue using 27.B1 (A, B, C) and 27.F7 (D, E, F) antibodies. Normal ovary: the epithelial monolayer is negative (A, D) (magnification ×400), borderline tumors (B, E) (magnification ×200) show different staining (27.B1 – positive, 27.F7 – negative). Epithelial serous carcinoma (C, F) is positive for both antibodies (magnification ×400). Black arrows indicate the negative staining and red arrows indicate the positive staining of the epithelium.

Following the results obtained with different cancer cell lines, we have extended our study by further analysis of 27.B1 and 27.F7 antibodies interaction with different types of malignant and benign breast tumors in order to evaluate the incidence of GIPC1 enhanced expression within a given tumor type and to compare GIPC1 expression in different breast tumor types. Different breast tissues including two benign entities – fibroadenoma and hyperplasia of the breast, and four malignant tumors – lobular carcinoma *in situ*, ductal carcinoma *in situ*, invasive lobular carcinoma and invasive ductal carcinoma (invasive and metastatic type) were examined. Immunoreactivity of 27.B1 and 27.F7 antibodies was observed only in invasive ductal (IDC) and invasive lobular carcinoma (ILC) (Figure 4-A and Table 1), in contrast to hyperplasia, fibroadenoma, lobular carcinoma *in situ *(LCIS) and ductal carcinoma *in situ *(DCIS) that demonstrated no staining at all (Table 1).

**Table 1:** IHC staining results for 27.B1 and 27.F7 in various breast lesions.

***Tumor type***	***Positive cases/total cases***	
	**27.B1**	**27.F7**
Hyperplasia	0/4	0/4
Fibroadenoma	0/4	0/4
LCIS (Lobular carcinoma in situ)	0/4	0/4
DCIS (Ductal carcinoma in situ)	0/4	0/4
ILS (Invasive lobular carcinoma)	9/10 (90%)	8/15 (53%)
IDC (Invasive ductal carcinoma)	24/25 (96%)	11/23 (48%)

Table 1 summarizes the results obtained from the immunohistochemistry of the breast lesions and illustrates the difference in staining percentage (positive cases out of the total number of samples studied) between different tumor types, implying differential incidence of GIPC1 enhanced expression.

According to our findings (Table 1), 27.B1 displays a relatively higher reactivity with breast tumors, detecting a greater percentage of cases upon the examination of each tumor type. In contrast, 27.F7 displays a lower reactivity, staining a lower percentage of tumor cells in the positive cases. Statistical examination of invasive ductal carcinoma (IDC) demonstrated significant difference between these antibodies (p < 0.001). Noteworthy, both antibodies did not stain benign tumors of the breast, nor normal controls. The 27.F7 and 27.B1 antibodies both display differential staining between various tumor types. Interestingly, GIPC1 staining with both 27.F7 and 27.B1 antibodies was positive in invasive breast cancer but not in in-situ carcinomas (p < 0.001).

Following the experiments with breast cancer, we decided to evaluate the possible association of 27.B1 and 27.F7 antibody staining with ovarian cancer tissue obtained from patients with benign cystadenoma, borderline tumor and the most aggressive ovarian malignancy-serous carcinoma of the ovary.

### 27.F7 and 27.B1 antibodies detect GIPC1 in ovarian serous carcinoma and shows differential staining of borderline tumors

Three types of ovarian tumors were examined using normal ovarian tissue as a negative control. Benign ovarian serous cystadenoma, serous cystadenoma of uncertain malignant potential (borderline malignancy) and the overtly malignant ovarian serous adenocarcinoma were all examined using the same immunohistochemical technique. The results demonstrate different staining for 27.F7 and 27.B1 antibodies in normal, benign and malignant ovarian epithelium. These antibodies also revealed differential staining of borderline tumors, displaying different percentage of positive cases, compared to benign and malignant tumors. Noteworthy, a correlation between positive staining and tumor invasiveness was observed (Table 2), which is similar to the breast cases. This implies a difference in GIPC1 over-expression between benign and malignant tumors, independent of the tissue type. The percentage of positive cases for 27.B1 antibodies correlated with increasing malignancy of the ovarian tumors. Overall, a similar correlation was observed for the 27.F7 antibody as well, although it was negative in the borderline tumors.

**Table 2:** IHC staining results for 27.B1 and 27.F7 in various ovarian tissues.

***Tumor type***	***Positive cases/total cases***	
	**27.B1**	**27.F7**
Normal ovary	0/8	0/8
Benign serous cystadenoma	2/15 (14%)	3/15 (21%)
Serous borderline tumor	4/11 (36%)	0/11
ESC (Epithelial serous carcinoma)	7/13 (54%)	8/15 (53%)

Difference between the number of GIPC-positive cases detected by 27.B1 and 27.F7 antibodies was significant (p < 0.045) in borderline tumor only. Table 2 summarizes the results obtained from the immunohistochemistry of the ovarian tissues and illustrates the difference in staining percentage (positive cases out of the total number of samples studied) between different tissues, implying differential incidence of GIPC1 enhanced expression.

According to our findings, 27.F7 and 27.B1 antibodies recognize ovarian cancer specifically, revealing the highest number of positive cases (more than 50%) in serous carcinoma (Table 2). On the other hand, the normal control remains negative, indicating a very high specificity of these antibodies to the malignant tumor. Both antibodies demonstrated similar reactivity with the malignant serous carcinoma, and thus, similar ability to detect elevated levels of GIPC1 protein in this type of ovarian tumor. Figure 4-B illustrates immunohistochemical staining of ovarian tumors using 27.F7 and 27.B1 antibodies.

Examination of borderline ovarian tumors (proliferative epithelial tumors with low risk of recurrence and metastases) showed that only 27.B1 antibody was reactive against this type of tumor, while 27.F7 was completely negative (Table 2). This difference was statistically significant (p < 0.045). According to these findings, 27.B1 and 27.F7 antibodies display differential staining of borderline tumors, suggesting different epitope recognition on the GIPC1 antigen, which is in agreement with previous results (Rudchenko et. al., Manuscript in press BMC cancer). The percentage of positive cases found in benign and borderline tumors is also lower than in overtly malignant serous carcinoma, suggesting a lower incidence of enhanced GIPC1 expression in these tumor types. However, only the increased immunoreactivity for 27.F7 antibody in malignant tumors compared to borderline tumors was statistically significant (p = 0.007).

All of the above findings suggest the next logical step in this study: identification of a correlation between positive tissue staining (high incidence of enhanced expression of GIPC1 in malignant tumors) and cancer specific autoantibody levels in ovarian and breast patients' sera targeting the GIPC1 antigen. According to this working hypothesis, the detection of these circulating anti-cancer specific antibodies using the cancer-associated GIPC1 antigen might be a sensitive marker for certain early stage malignancies and superior to methodologies based on cancer-associated antigen detection. As a result, our next goal is the development of a novel screening technique based on sensitive detection of cancer specific autoantibodies in patients' sera instead of searching for circulating tumor antigen, which is more commonly performed in conventional screening techniques today.

## Discussion

In this pilot study we determined that the reactivity of fully human monoclonal antibodies (fhMAbs) 27.B1 and 27.F7, derived from lymph node B-cells of a breast cancer patient and targeting the PDZ domain-containing protein known as GIPC1, are specific to breast and ovarian cancer. Through exploration of the interaction of 27.F7 and 27.B1 autoantibodies with breast cancer cell lines SKBR-3, MCF-7 and ovarian cancer cell line SKOV-3, we found a very strong signal in staining of breast, ovarian and pancreas cancer cell lines, but not in normal controls. The results of these experiments reveal that these two anti-GIPC1 human monoclonal antibodies bind to cancer cells specifically but are not limited to binding a single malignant cell type. Instead they have broader cancer reactivity than we previously thought. Moreover, flow cytometry has demonstrated that the reactivity of 27.F7 antibody with the SKBR-3 cell surface is significantly stronger than that of 27.B1. Furthermore, we have also demonstrated that these antibodies not only bind to the surface of cancer cells, but are internalized, in contrast to control immunoglobulin. As such, these antibodies may impact directly on GIPC1 intracellularly, and therefore may affect a variety of different cancer-associated signaling pathways. Therefore, these antibodies may be useful not only diagnostically but also therapeutically in the future.

These results provided the impetus for our present immunohistochemical studies, and further examination of 27.B1 and 27.F7 human monoclonal antibodies can provide a base for their potential future applications in immunohistochemical research, cancer screening and diagnostics.

We also studied the interactions of 27.F7 and 27.B1 antibodies with human breast and ovarian tumor tissue under the working hypothesis that these two human monoclonal antibodies target different GIPC1 epitopes. The immunohistochemical studies of 27.F7 and 27.B1 have also enabled a comparison of GIPC1 levels in different types of breast and ovarian tumors. Our results clearly demonstrate elevated levels of GIPC1 in malignant, but not benign breast tumors. Therefore, we suppose that this protein is cancer-associated, and might play a role in a cancer-associated process. Another potential explanation that might be suggested is that malignant processes taking place in the proliferating cells require an increased amount of GIPC1 protein, enabling its detection in immunohistochemical staining.

Analysis of ovarian tumors with 27.F7 and 27.B1 antibodies demonstrated differential staining between them, which was statistically significant for borderline ovarian tumors, displaying exclusive binding of 27.B1, but not 27.F7 antibody.

Based on all of our immunohistochemical findings we hypothesize that the antibodies target different epitopes. This is also supported by the fact that they display differential binding upon examination of invasive malignant tumors, and also with respect to borderline ovarian tumors. Since our immunohistochemical results were obtained in a pilot study, this finding requires further investigation on a larger sampling to clarify if this might be used as cancer diagnostic and prognostic tool.

Benign, borderline and malignant conditions are different in terms of their gross morphology and fine structure and are expected to be associated with different cellular processes, perhaps leading to different protein distribution and compartmentalization. The conformation of a cellular protein might also change according to its function at a given moment and in a given tissue implying different epitope accessibility, which might explain in part the differential staining obtained by 27.B1 and 27.F7 antibodies. Nonetheless, the reason for such a difference between the sensitivities of these antibodies to the GIPC1 protein in the tissues that were examined still remains unclear. Recently, it has been shown that GIPC1 can form multimers by binding to itself and that the PDZ domain is involved in the GIPC-GIPC interaction. Furthermore, it was shown that whereas the bulk of cytosolic GIPC1 was present as monomer, GIPC1 homotrimer was readily detectable in the membrane fraction [21]. These results support our findings and help explain the differential tissue staining obtained by 27.B1 and 27.F7 antibodies.

The results obtained from the examination of benign ovarian cystadenoma, borderline ovarian tumor and invasive ovarian serous carcinoma demonstrate a direct correlation between tumor malignancy and staining. It can be clearly seen that the highest number of GIPC1 positive cases was found for epithelial serous carcinoma – a malignant tumor of the ovary. The behaviour of borderline ovarian tumors is uncertain; they usually behave in a benign fashion, but they have a potential for recurrences in the form of peritoneal implants or even as a metastatic disease. With this in mind, along with other results, we developed a few hypotheses regarding the possible cause for significantly elevated levels of GIPC1 detected in malignant and borderline ovarian tumors. We propose that GIPC1 overproduction is not a direct cause of cancer, as mentioned above, but is rather a byproduct of the cellular response to the transformed state. In addition, it could be evidence for an attempt by the cell to balance and regulate itself.

Regardless of the role that GIPC1 might play in carcinogenesis, it is clearly a novel cancer-associated antigen, suggesting that further research should be conducted in order to define its role in cell transformation and cancer development. To further support the correlation between GIPC1 expression and clinical pathologies, a full-scale study is being performed. Our study, however, forms the basis for further broad-based research of the differential interaction of the fully human 27.B1 and 27.F7 autoantibodies with GIPC1 antigen in different malignant and benign tumors, which will likely be useful for research and diagnostics. Further investigations of the role these antibodies play in the progression of cancer, and the role of GIPC in cancer, is therefore warranted.

## Competing interests

The authors declare that they have no competing interests.

## Authors' contributions

VY and AT performed many of the experiments and prepared much of this manuscript. SR, TA and HK performed immunocytochemcial and immunohistochemcial experiments. RS–L and BD are pathologists at Soroka Hospital who performed immunohistochemical analysis and chose tissue blocks for these studies. AR and BP provided patient specimens and helped prepare the manuscipt. IT and LL initiated these studies, designed most of the experiments and wrote this manuscript.

## Pre-publication history

The pre-publication history for this paper can be accessed here:


